# Functional Implications of Human-Specific Changes in Great Ape microRNAs

**DOI:** 10.1371/journal.pone.0154194

**Published:** 2016-04-22

**Authors:** Alicia Gallego, Marta Melé, Ingrid Balcells, Eva García-Ramallo, Ignasi Torruella-Loran, Hugo Fernández-Bellon, Teresa Abelló, Ivanela Kondova, Ronald Bontrop, Christina Hvilsom, Arcadi Navarro, Tomàs Marquès-Bonet, Yolanda Espinosa-Parrilla

**Affiliations:** 1 IBE, Institute of Evolutionary Biology (Universitat Pompeu Fabra-CSIC), Department of Experimental and Health Sciences, Barcelona, Catalonia, Spain; 2 CRG, Center for Genomic Regulation, Barcelona, Catalonia, Spain; 3 Department of Stem Cells and Regenerative Biology, Harvard University, Cambridge, MA, United States of America; 4 Broad Institute of MIT and Harvard, Cambridge, MA, United States of America; 5 Department of Biosciences, Viikki Biocenter, University of Helsinki, Helsinki, Finland; 6 Parc Zoològic de Barcelona, Barcelona, Catalonia, Spain; 7 Universitat Autònoma de Barcelona, Bellaterra, Catalonia, Spain; 8 Biomedical Primate Research Centre, Rijswijk, Netherlands; 9 Copenhagen Zoo, Frederiksberg, Denmark; 10 INB, National Institute for Bioinformatics, Barcelona, Catalonia, Spain; 11 ICREA, Catalan Institution for Research and Advanced Studies, Barcelona, Catalonia, Spain; 12 CNAG, Centro Nacional de Análisis Genómico, Barcelona, Catalonia, Spain; 13 School of Medicine, University of Magallanes, Punta Arenas, Chile; University of Texas, MD Anderson Cancer Center, UNITED STATES

## Abstract

microRNAs are crucial post-transcriptional regulators of gene expression involved in a wide range of biological processes. Although microRNAs are highly conserved among species, the functional implications of existing lineage-specific changes and their role in determining differences between humans and other great apes have not been specifically addressed. We analyzed the recent evolutionary history of 1,595 human microRNAs by looking at their intra- and inter-species variation in great apes using high-coverage sequenced genomes of 82 individuals including gorillas, orangutans, bonobos, chimpanzees and humans. We explored the strength of purifying selection among microRNA regions and found that the seed and mature regions are under similar and stronger constraint than the precursor region. We further constructed a comprehensive catalogue of microRNA species-specific nucleotide substitutions among great apes and, for the first time, investigated the biological relevance that human-specific changes in microRNAs may have had in great ape evolution. Expression and functional analyses of four microRNAs (miR-299-3p, miR-503-3p, miR-508-3p and miR-541-3p) revealed that lineage-specific nucleotide substitutions and changes in the length of these microRNAs alter their expression as well as the repertoires of target genes and regulatory networks. We suggest that the studied molecular changes could have modified crucial microRNA functions shaping phenotypes that, ultimately, became human-specific. Our work provides a frame to study the impact that regulatory changes may have in the recent evolution of our species.

## Introduction

Over the last years, attention has been focused on the role that regulatory elements may have played in shaping diversification among species and individuals that share extended genomic similarities. Certain specific changes in transcription factors and non-coding RNAs have been shown to be under positive selection and to contribute to determine phenotypic differences between species such as humans and chimpanzees [1,2]. Among non-coding RNAs, microRNAs (miRNAs) are key post-transcriptional gene regulators with a clear role in evolution that are implicated in almost every biological function and in many types of diseases such as cancer and neurological disorders [3–5].

miRNAs are initially transcribed by RNA polymerase II as primary miRNAs, from which Drosha-mediate cleavage gives a ~70 nt hairpin precursor miRNA (pre-miRNA), that is processed by Dicer in the cytoplasm into a miRNA duplex formed by two ~22 nt mature miRNA strands, 5p and 3p [6]. One of the strands is then incorporated into the Argonaute-containing miRNA-induced silencing complex (RISC), that guides the miRNA to its target messenger RNA (mRNA) inhibiting its expression either by translation repression or mRNA degradation [7,8]. Target gene repression is based on partial complementarity between the miRNA and the mRNA, but a perfect match between the seed region of the miRNA (positions 2–8 of the mature miRNA) and the target site on the mRNA is needed [9]. Initially, it was believed that only one of the two mature miRNA strands, named the major strand, played a significant role in gene regulation, while the other one was degraded. Recent studies, however, stress out the biological importance of both mature strands in gene repression [8,10,11].

miRNAs can be located either in intergenic regions or within genes, and they can be organized as single miRNAs or as clusters containing several miRNAs, which are usually transcribed together in a polycistronic primary miRNA [8,12]. Most human miRNA clusters contain from two to eight miRNAs but over 40 miRNAs have been reported in two clusters on chromosomes 14 and 19 [13].

Previous studies have shown that the miRNA repertoire has constantly increased during metazoan evolution. It is estimated that, in mammals, half of the new-born miRNAs would be deleterious and eliminated by purifying selection; however, some of them become integrated into gene regulatory networks having low chances to be subsequently lost [10,14–16]. The emergence rate of miRNA has varied over evolutionary time. During mammalian evolution, the net gain of miRNA families was three times higher in therians (marsupials and placental mammals) than in monotremes and birds [16] and about a quarter of the total known human miRNAs seem to have appeared at the beginning of the hominoid lineage [17]. These miRNA expansions have been associated with evolutionary innovations that, for example, lead to the diversification of bilaterians, vertebrates and mammals, consistent with the existing correlation between the number of miRNAs and organism complexity [10,14,18].

So far, the study of primate miRNAs has been mostly focused on the identification of orthologous miRNAs in different species. Firstly by comparative genomic approaches taking advantage of the high level of sequence conservation of miRNA genes across species [19–21]; and more recently by small RNA sequencing of different tissues in various species [16,22,23]. Among primates, humans are the deepest explored group with 1,881 pre-miRNAs described, followed by chimpanzees with only around six hundred pre-miRNAs already identified [24]. Two recent studies have found numerous novel human miRNAs: 2,469 [25] and 3,500 [23], not yet included in miRBase but increasing the gap between human and non-human primate miRNA annotations.

It has been shown that selective pressures have differentially affected miRNA regions in animal evolution. The seed region has been commonly considered as being the most conserved region of miRNAs as well as the main region for target gene recognition and consequently for gene regulation. Nevertheless, increasingly number of studies has shown that other positions along the mature region could be under similar selective constraint as demonstrated by a research in metazoans showing that the seed region and nucleotides 13 to 16 of the mature molecule are the most conserved miRNA nucleotides [10]. This agrees with observations of variation across different human populations that show purifying selection has globally constrained mature miRNA sequences, specifically the first 14 nucleotides including the seed region [26–28]. However, the possibility that lineage-specific changes in miRNAs may have contributed to shape species phenotypic differences has not been addressed. In the present study, we took advantage of a recently published set of genomes from ten great ape populations [29] to systematically identify all substitutions that occurred in miRNAs along the great ape phylogeny. By comparing patterns of intra- and inter-species variation, we explored the strength of selection in different miRNA regions and in miRNA groups classified based on different genomic and functional characteristics. Finally, we focused on the functional characterization of four miRNAs (miR-299-3p, miR-503-3p, miR-508-3p and miR-541-3p) that contain human-specific changes in the mature region. We found that human-specific substitutions in these miRNAs together with length modifications have altered their expression levels and their repertoire of target genes indicating that human-specific changes in miRNAs may have played a crucial role in fine-tuning regulatory networks that, ultimately, have become human-specific.

## Materials and Methods

### Human miRNA classification and definition of miRNA regions

Genomic coordinates of 1,595 human pre-miRNAs containing 2,233 mature miRNAs were obtained from miRBase (release 19). Nucleotides 2 to 8 of the 5’ end of mature miRNAs were defined as the seed region. We considered flanking 5’ and 3’ regions of 200 bp upstream and downstream of the pre-miRNA, respectively. We defined miRNAs as clustered when they were located less than 8 Kb from another miRNA and as isolated otherwise. We classified miRNAs as intragenic when they overlapped an annotated RefSeq gene and as intergenic otherwise. Intragenic miRNAs were further classified as intronic, exonic or non-coding. We also grouped miRNAs regarding their molecular age [17,30] and the number of diseases they were involved in (Human miRNA Disease Database) [31]. Expression data of miRNAs in human brain came from Allen Brain Atlas [32].

### Great ape sequence dataset

Data was taken from Prado-Martinez et al. [29] where 82 great apes were genome sequenced at high-coverage and all reads were mapped against the human genome (hg18). We discarded one individual *Gorilla gorilla dielhi* from [29] data since it was the only one from this population (S1 Table). We masked uncallable regions and recent repetitive regions defined by repeat masker [33] excluding those repeats shared between humans and rodents. To orientate all substitutions across the phylogenetic tree we used ancestral information [29].

### Divergence and diversity calculations

For the inter-species variation analyses, we used the density of single nucleotide variants (SNV density) fixed in at least one of the populations along the phylogeny as a surrogate measure of purifying selection. Given that the time of divergence between the great ape populations is relatively small, we assumed the number of fixed substitutions along the great ape phylogeny to be proportional to the substitution rate. We excluded all positions that were polymorphic in at least one great ape population (1000 Genomes database) [34]. We used a generalized linear model to test whether the different regions of the miRNAs had differences in their SNV density (normalized using the arcsine and taking into account GC content). For the intra-species analyses, we calculated nucleotide diversity [35] as a measure of intra-species diversity for all regions of the concatenated miRNAs. To be able to compare across regions, rather than across populations, we standardized the value of nucleotide diversity using Z-scores. PhastCons conservation scores for primates were downloaded from UCSC [36]. Average for the different regions of the miRNA was calculated for each gene. Uncallable regions were masked and SNP density and nucleotide diversity were calculated for each miRNA region.

### Principal Component Analysis (PCA) and correlation analyses

The PCA was performed using the prcomp function in R. The variables used in the PCA were SNV density for the pre-miRNA and other miRNA features such as miRNA clustering, expression levels, involvement in disease, molecular age and localization regarding other transcriptional units. The 576 miRNAs for which these features were available were included in the PCA. The correlation study between the expression data and the SNV density included miRNA gene expression from five human tissues (brain, cerebellum, heart, testis and kidney) [16]. We used the mean expression across tissues to assess the relationship between conservation and expression. To calculate specificity we used the coefficient of variation.

### Ethics statement

This work was conducted according to relevant Spanish and International guidelines. Tissue samples were collected after natural death and thus it was done in a non-invasive way without disturbing, threatening or harming the animals. Blood samples were explicitly not taken for this study.

### DNA and RNA samples

Gorilla brain tissue was provided by the Barcelona Zoo (Spain). Chimpanzee and macaque brain and testis tissues were provided by the Biomedical Primate Research Centre (Netherlands). Total RNA was extracted with the RNeasy Mini Kit (QIAGEN, Valencia, CA, USA). DNA was extracted using the EZNA^®^ Tissue DNA Kit from Omega BioTek (Norcross, GA, USA). Mouse brain RNA and human RNA samples were purchased at Ambion (Foster City, CA, USA).

### miRNA cloning and transfection experiments

For expression level analyses, pre-miRNA genomic sequences of the selected miRNAs (mir-299, mir-503, mir-508 and mir-541) and ~100 bp flanking regions at each side were PCR amplified (primers in S2 Table). PCR fragments were cloned into the pmR-ZsGreen1 vector (Clontech) through *BamH1* and *XhoI* restriction sites and transfected in HeLa cells grown as previously described [37] scaled to 1.8x10^5^ cells/well in 12-well plates. After 24h, cells were double-transfected with Lipofectamine 2000 (Invitrogen) and 0.75 μg of DNA with the plasmid constructions carrying either the human or non-human pre-miRNAs plus a control reference miRNA. Transfections were optimized to an efficiency of over 70% by monitoring the expression of the fluorescent ZsGreen1 protein. Three independent experiments each including three technical replicates were performed. For transcriptome analyses SH-SY5Y cells were transfected with 100 μM of miRNA mimic variants (miR-299-3p, miR-503-3p, miR-508-3p, miR-541-3p or the related negative control) as previously described [38]. A fluorescently labeled mimic control (miRIDIAN^™^ miRNA Mimic Transfection Control with Dy547, Dharmacon) was used to optimize the transfection to an efficiency of over 70%. Four independent experiments were performed. In both approaches non-human miRNA sequences were taken from chimpanzee, except for mir-541, for which we took the macaque sequence since no chimpanzee mir-541 has been described so far.

### Expression analysis by real time quantitative reverse transcription PCR (RT-qPCR)

miRNA expression analyses were performed by RT-qPCR starting from 100–300 ng of total RNA from either different mammalian tissues (for measuring tissue expression) or transfected HeLa cells (for measuring expression level differences) using specific primers for each species variant (S2 Table). Primers design and RT-qPCR protocol were performed as previously described [39]. The qPCR was performed using LightCycler^®^ 480 SYBR Green I Master (Roche Diagnostics) following manufacturer’s protocol. Standard curves were calculated separately for each miRNA variant by pooling all samples from transfection experiments. Expression level comparisons between human and non-human miRNA variants were calculated from RT templates 20x diluted using the method published by Pfaffl [40] by quantifying the relative expression ratio of the studied miRNA based on the efficiency and the quantification cycle deviation (ΔCq) between the two miRNA variants (human and non-human), and comparing versus a control reference miRNA. Expression of miR-25-3p was analyzed as a control reference miRNA gene across mammalian tissues showing similar Cq values (ranking from 19.6 to 21.2) in all tissues.

### Whole-genome expression microarrays analyses

RNA samples from four independent transfection experiments of each miRNA variant in SH-SY5Y cells were used for microarray expression experiments in Agilent SurePrint G3 Human Gene Expression microarrays (8x60k) starting from 300ng of total RNA. Data were analyzed using the Array File Maker (AFM) 4.0 software package (Array File Maker, Stanford, California). We considered genes differently expressed when comparing human and non-human miRNA mimic variants (p < 0.05; fold change > 1.2) and when comparing each variant with the negative mimic control (adjusted p < 0.05; fold change > 1.2). Analyses on the biological functions associated with deregulated genes were performed using the Ingenuity Pathway Analysis software (IPA). Microarray data is publically available at the ArrayExpress database (www.ebi.ac.uk/arrayexpress) under accession number E-MTAB-3617.

### miRNA target gene prediction

miRNA target gene predictions were made using “Probability of Interaction by Target Accessibility” algorithm (PITA) [41]. In order to identify possible target genes for each miRNA variant we used all described 3’ UTRs in human genes (RefSeq). A gene was considered as a predicted target if the minimum accessibility energy score (ΔΔG) was ≤ -10 (the recommended cutoff) [41], and as an exclusive target gene that is only regulated by one of the two variants if ΔΔG ≤ -10 for one variant and the difference between variants ≥ 3 or if ΔΔG ≤ -8 and that difference ≥ 4. Predicted target genes in non-human primate genomes were confirmed by searching at their reference genomes (panTro4 for chimpanzee, rheMac3 for macaque).

### Secondary structure predictions of miRNAs

Minimum free energy (MFE) of the secondary miRNA structures were based on RNAfold predictions. miRNA sequences were taken from miRBase (release 21). Non-human miRNA sequences were taken from chimpanzee, except mir-541 that was taken from macaque.

## Results

### The seed and mature miRNA regions are equally conserved in great apes

**Fig 1:**
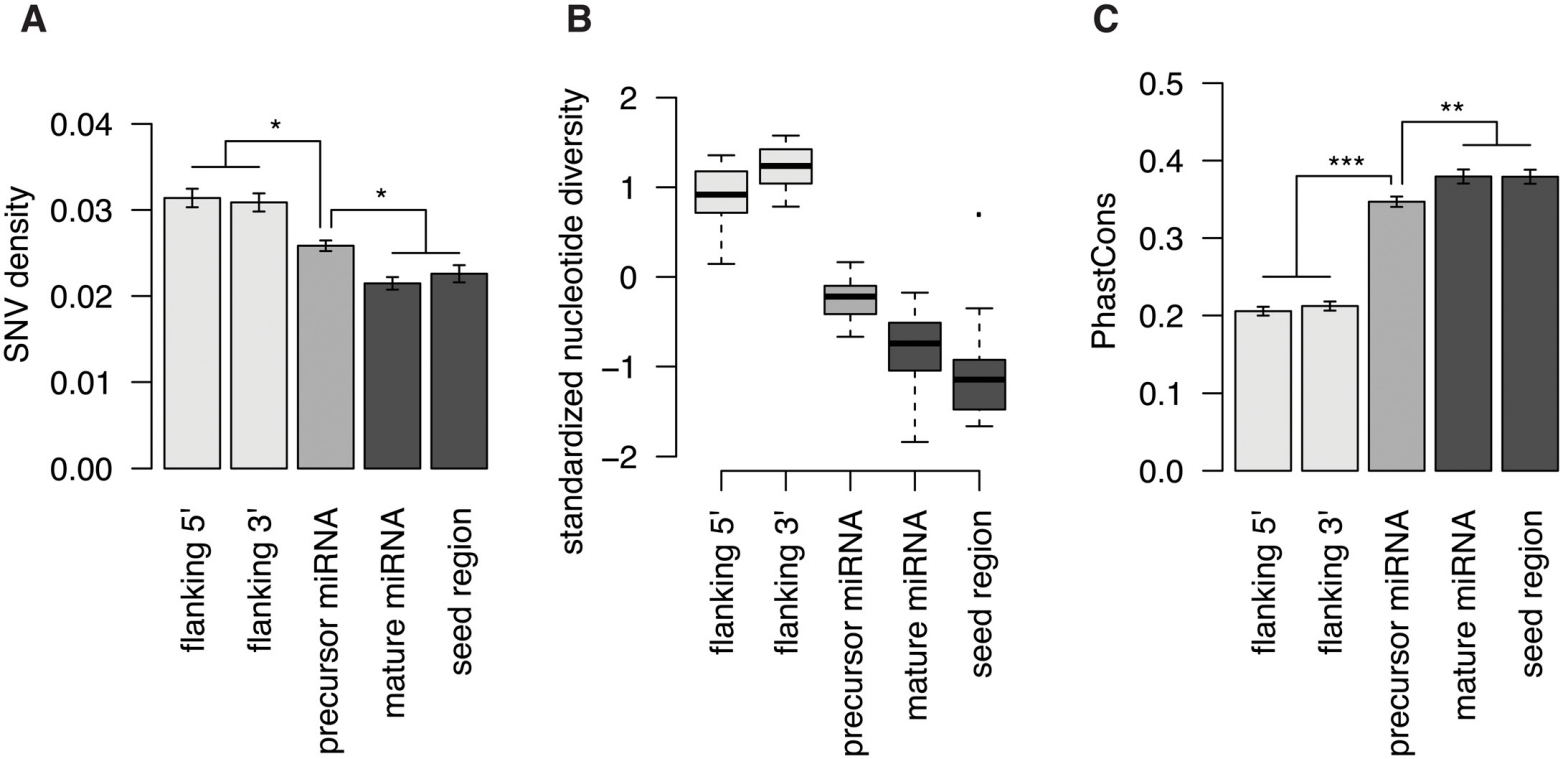
Variation across miRNA (precursor, mature and seed) and flanking (3’ and 5’) regions, in the great ape populations. (**A**) Single nucleotide variant (SNV) density calculated as the average number of fixed substitutions in concatenated regions within each population. (**B**) Boxplot showing standardized (Z-score) nucleotide diversity (pi). (**C**) Average PhastCons conservation scores. Regions with no statistically significant differences share the same color. Asteriks represent statistically significant differences. Error bars in (**A**) and (**C**) represent the standard error of the mean.

To systematically study the recent evolutionary history of miRNAs we used high-coverage sequencing data from 82 individuals from ten great ape populations including humans (S1 Table). We first looked for differences in sequence conservation among miRNA functional domains by calculating single nucleotide variants density (SNV density) in 1,125 out of 1,595 human miRNAs (miRBase, release 19) for which we found an orthologous miRNA gene in all great ape species. We calculated SNV density across the great ape phylogeny in the precursor (excluding the mature), mature (excluding the seed) and seed regions of the concatenated miRNA sequences as well as in their 5’ and 3’ flanking regions. As previously reported, the precursor, the mature and the seed regions were more conserved than the miRNA 5’ and 3’ flanking regions (generalized linear model, GLM; p < 0.05, Fig 1A, S3 Table). Surprisingly, conservation of the seed region was not significantly different from the mature miRNA, while both mature and seed were more conserved than the precursor miRNA region. Further, we looked at intra-specific variation using polymorphic variants inside each great ape population and calculating nucleotide diversity (Fig 1B). We observed similar patterns of conservation both in the intra- and inter-specific analyses.

To rule out the possibility that we did not observe differences between the seed and the rest of the mature miRNA due to short evolutionary distances in the analysis that do not provide enough information, we calculated average PhastCons conservation scores in each of the miRNA regions using pre-computed values for all primates [36]. In accordance with our previous results, mature miRNAs and their seed regions had nearly identical average conservation scores and both had higher conservation scores (p < 0.05) than the precursor and flanking regions (Fig 1C).

### miRNA conservation is mainly explained by their molecular age

**Fig 2:**
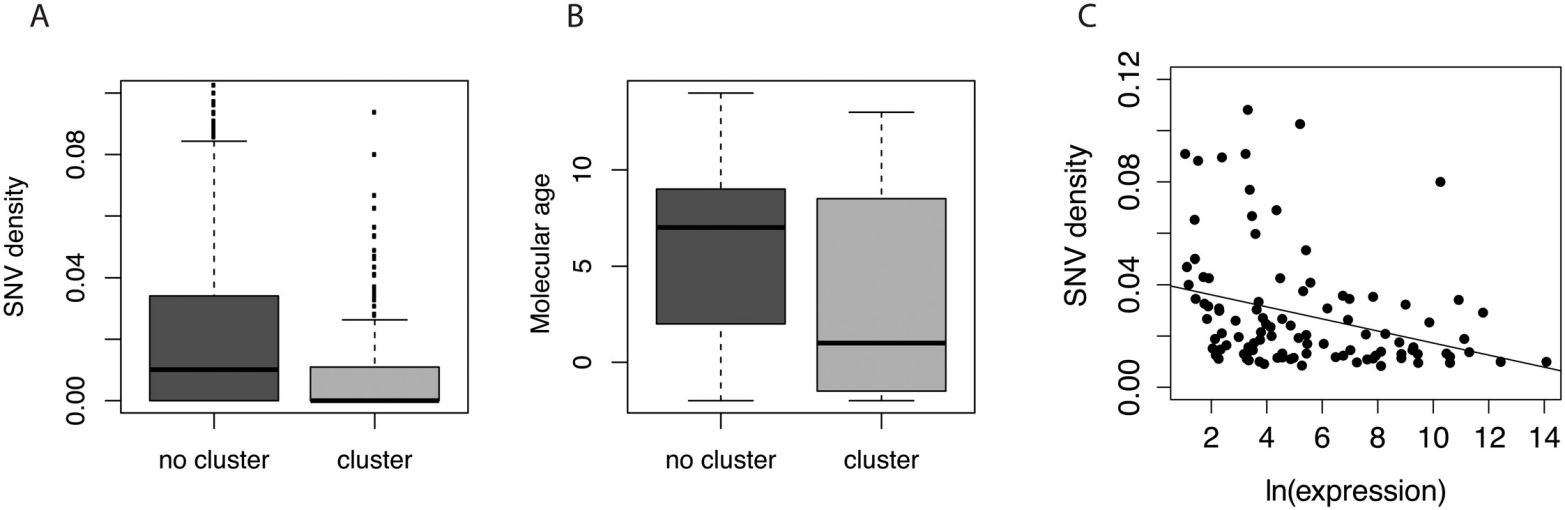
miRNA conservation in clustered and non-clustered miRNAs. (**A**) Single nucleotide variant (SNV) density in clustered or non-clustered miRNAs, calculated as the average number of fixed substitutions in any of the great ape populations across the precursor miRNA. (**B**) Molecular age of clustered and non-clustered miRNAs. Molecular age is taken from Iwama et al. [17] were each integer represents a period of origin with the oldest miRNAs having a value of -1 (right after the split between mammals and birds) and the youngest a value of 13 (after the split between humans and chimpanzees). (**C**) Correlation between SNV density and expression, calculated as the average expression values for miRNAs across five human tissues (cerebellum, brain, heart, kidney and testis) taken from Meunier et al. [16].

To assess whether any feature of the miRNAs, such as their levels of expression, their molecular age, their role in disease or their location respect to other transcriptional units, was correlated with their levels of constraint we ran a principal component analysis. The first component was mostly explained by the number of diseases each miRNA is involved in (data according to Human miRNA Disease Database) [31], whereas the second component was mostly explained by molecular age based on [30] and [17] estimations (S1 Fig). Indeed, more conserved miRNAs are older, involved in a larger number of diseases and often located in miRNA clusters. However, when we specifically tested which of these variables explained the substitution patterns of miRNAs, the only significant variable was molecular age (GLM; p < 0.001). Therefore, although miRNAs that belonged to clusters had lower SNV density (Fig 2A), when molecular age was considered in the model, differences between clustered and non-clustered miRNAs were lost (Fig 2B), indicating that the main determinant of miRNAs conservation is the time they originated. Finally, we evaluated whether there was a correlation between the expression patterns of miRNAs across five human tissues (cerebellum, brain, heart, kidney and testis) (data according to Meunier et al.) [16] and their sequence conservation (Fig 2C). We found that SNV density was negatively correlated with average miRNA expression levels (Spearman correlation; ρ = -0.27; p < 0.05) but not with tissue specificity (Spearman correlation; p > 0.05; data not shown).

### miRNAs with human-specific nucleotide substitutions are less expressed than miRNAs without changes

**Fig 3:**
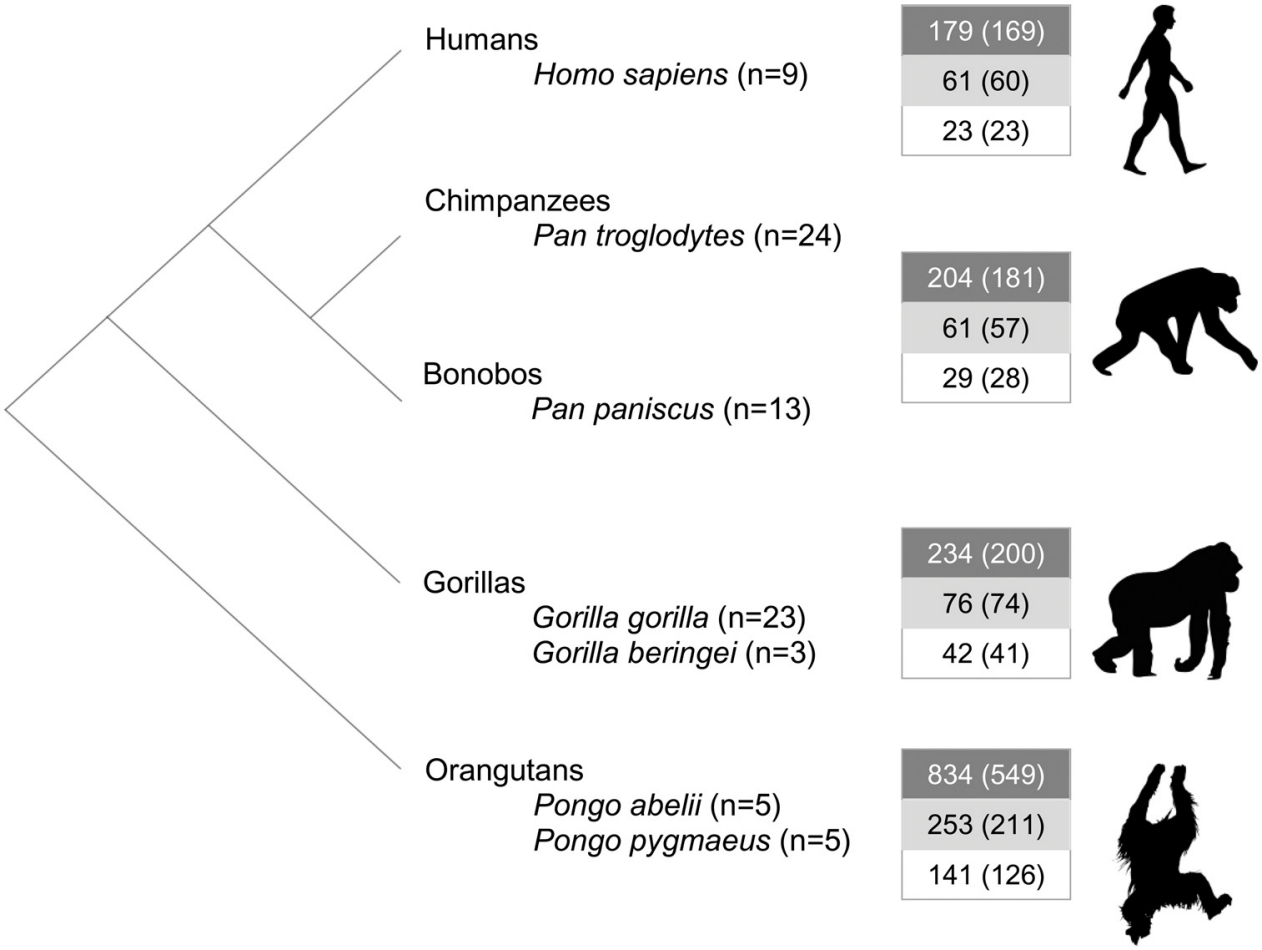
Great ape individuals and miRNAs changes analyzed in this study. Boxes indicate the number of species-specific nucleotide substitutions along the great ape phylogeny since the split with humans (or with chimpanzees in the case of humans), in the precursor (dark grey), mature (light grey) and seed (white) miRNA regions. Total number of miRNAs in which these changes occur is shown in brackets. No species-specific nucleotide substitutions were considered for bonobo (*Pan paniscus*) due to the low quality genome annotation in this group may underestimate the real number of species-specific substitutions in the rest of the groups, and for gorillas (*Gorilla beringei*) due to the low number of individuals that are representative of this population.

Identifying miRNA species-specific substitutions along the great ape phylogeny could provide new insights into the impact these changes may have had in the recent evolutionary history of great apes. We calculated the number of fixed nucleotide substitutions in miRNAs along the different great ape populations (Fig 3) and found that, in humans, 235 pre-miRNAs accumulated 263 human-specific substitutions (179 in the precursor excluding the mature sequence, 61 in the mature excluding the seed and 23 in the seed). Only 26 miRNAs had more than one fixed substitution with a maximum of three in two miRNAs (hsa-mir-614 and hsa-mir-3909, S4 Table). When comparing the 235 miRNAs carrying substitutions fixed in humans with the rest, we observed that miRNAs with human-fixed substitutions were mostly isolated, less duplicated, less associated with disease, younger (Chi-square test; p < 0.05) and had lower conservation values (Phastcons, t-test; p < 0,05). Next, we compared expression levels between pre-miRNAs carrying or not substitutions fixed in humans using expression data from the five human tissues mentioned above. We observed that miRNAs with substitutions fixed in humans (n = 20) were significantly less expressed in cerebellum, heart and kidney than miRNAs without changes (n = 266) (t-test; p < 0.05), particularly when only miRNAs with changes within the mature region (n = 6) were considered. In this latter case, expression differences were also statistically significant in brain but not in testis.

**Fig 4:**
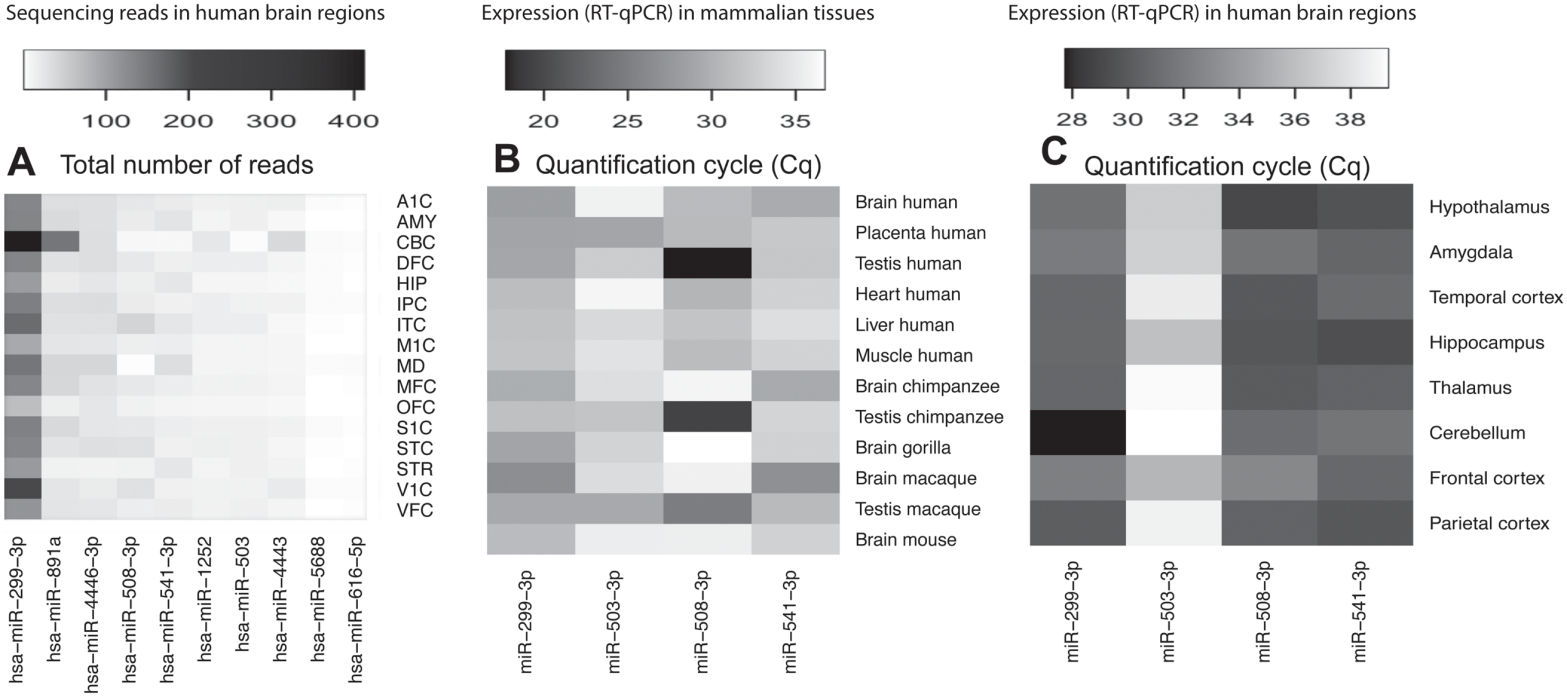
miRNA expression levels. (**A**) Expression levels represented as total number of sequencing reads of the ten miRNAs with human-specific substitutions in the mature or seed region and with the highest expression levels across brain regions (values taken from Allan Brain Atlas) [32]. (**B**-**C**) Expression levels of miR-299-3p, miR-503-3p, miR-508-3p and miR-541-3p measured by RT-qPCR in different mammalian tissues (**B**) and in different human brain regions (**C**). Brain regions in (**A**) are A1C: primary auditory cortex (core); AMY: amygdaloid complex; CBC: cerebellar cortex; DFC: dorsolateral prefrontal cortex; HIP: hippocampus (hippocampal formation); IPC: posteroinferior (ventral) parietal cortex; ITC: inferolateral temporal cortex (area TEv, area 20); M1C: primary motor cortex (area M1, area 4); MD: mediodorsal nucleus of thalamus; MFC: anterior (rostral) cingulate (medial prefrontal) cortex; OFC: orbital frontal cortex; S1C: primary somatosensory cortex (area S1, areas 3,1,2); STC: posterior (caudal) superior temporal cortex (area TAc); STR: striatum; V1C: primary visual cortex (striate cortex, area V1/17); VFC: ventrolateral prefrontal cortex. Brain tissues in (**B**) correspond to frontal cortex in all species.

Since mutations in the mature miRNA sequence are predicted to have a stronger impact on miRNA function than mutations in the precursor sequence we focused on the first as potential candidates for functional studies. We considered the 82 miRNAs with human-specific nucleotide substitutions in the mature region and selected the miRNAs with the highest average expression levels in brain, a tissue with high levels of miRNA regulation activity [5], based on Allen Brain Atlas database [32] (Fig 4A). After discarding polymorphism changes (1000 Genomes database) [34], four miRNA candidates were studied: miR-503-3p, with a single human-specific nucleotide substitution in the seed region, and miR-299-3p, miR-508-3p and miR-541-3p with human-specific nucleotide substitutions in the mature out of the seed region (Table 1). We then analyze their expression patterns in a set of mammalian tissues by RT-qPCR (Fig 4B). We found expression of miR-299-3p in brain, placenta and testis in all the studied primates and mouse. Interestingly miR-508-3p was expressed in human brain but almost undetectable in the brain of other primates or in mouse, although it had high expression levels in all primate testes. miR-541-3p was mostly expressed in human and non-human primate brain, while miR-503-3p was not amplified in brain and its expression was specifically found in human placenta and macaque testis. To better characterize the specific expression of these miRNAs in the central nervous system, we measured their expression levels in different human brain regions. Remarkably, miR-299-3p was highly amplified in cerebellum but poorly detected in other brain regions whereas miR-508-3p and miR-541-3p were expressed in several regions such as hypothalamus, hippocampus, frontal, parietal and temporal cortex. Consistent with the earlier results, miR-503-3p was lowly expressed in all brain regions studied (Fig 4C).

**Table 1:** Main characteristics of the miRNAs selected for functional analyses.

	Chr (hg19)	Cluster / Isolated	Intergenic / Intragenic	Number of associated diseases^1^	Molecular age^2^	Reported tissues expression^3^	Number of human-specific nucleotide substitutions		
							Precursor	Mature	Seed
hsa-miR-299-3p	14	Cluster	Intergenic	5	Mammals	Brain, cerebellum, testis	0	1	0
hsa-miR-503-3p	X	Cluster	Intragenic (non-coding)	7	Mammals	Testis, kidney	0	0	1
hsa-miR-508-3p	X	Cluster	Intergenic	3	Mammals	Testis, kidney	1	1	0
hsa-miR-541-3p	14	Cluster	Intergenic	0	Mammals	Brain, cerebellum	0	1	0

^**1**^Number of diseases based on Human MicroRNA Disease Database (HMDD) [31], update 2013.

^**2**^Molecular age data from Iwama et al. [17].

^**3**^Expression data in human tissues from Meunier et al. [16].

### Molecular changes in the miRNAs are related to differences in their expression levels

**Fig 5:**
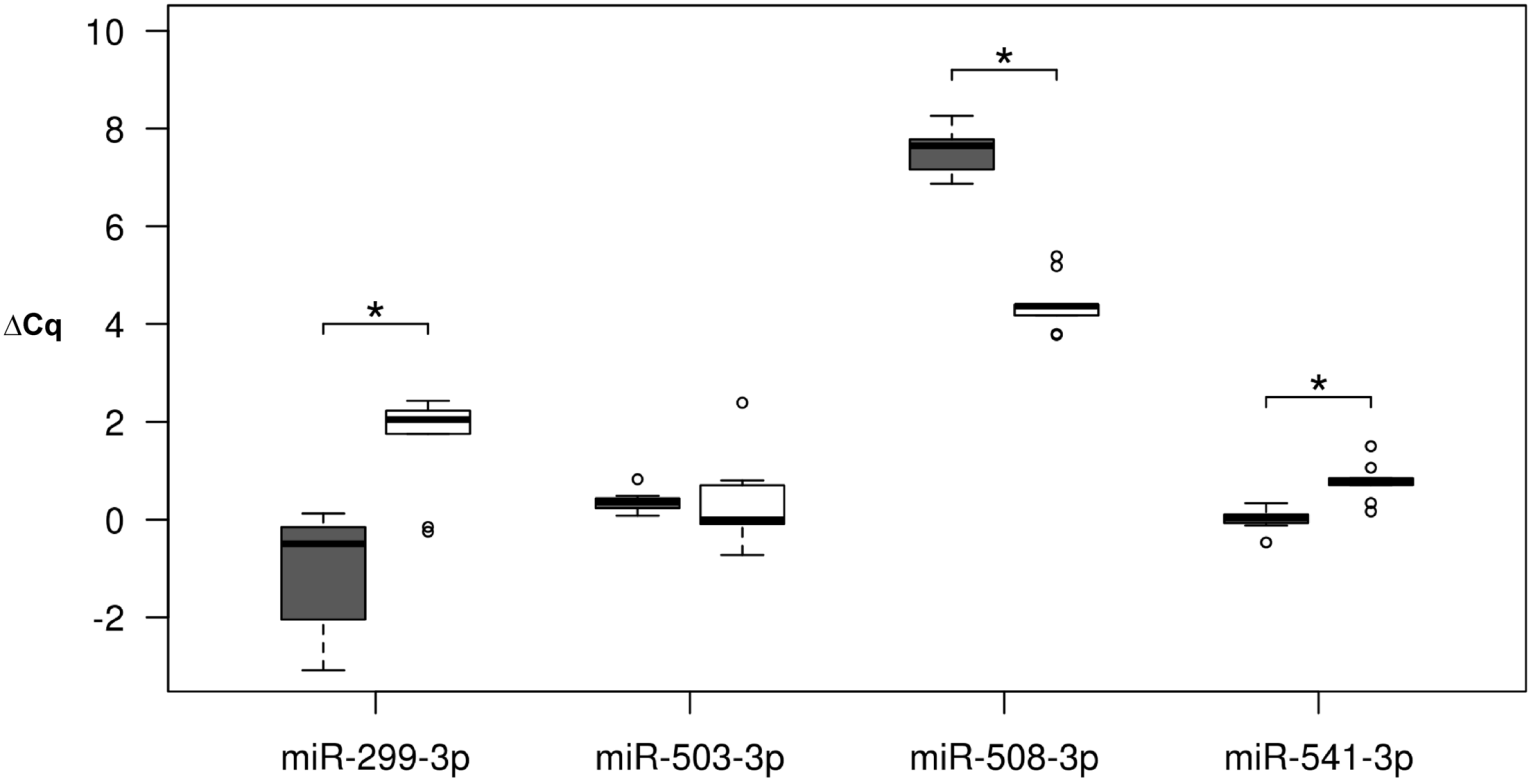
miRNA expression level differences between miRNA variants. Differences in the expression levels (ΔCq) measured by RT-qPCR in transfected HeLa cells for the studied miRNAs between the human (grey, left) and non-human (white, right) miRNAs. ΔCq represents the difference between the control reference miRNA and each miRNA variant. Asterisks indicate p < 0.05 in the t-test comparisons.

To test if human-specific substitutions in miRNAs could implicate differences in miRNA expression, we measured the expression of the four selected miRNAs in HeLa cells transfected with either the human or the non-human pre-miRNAs. Expression levels of the three miRNAs with nucleotide substitutions located in the mature out of the seed region were significantly different between the human and non-human miRNAs (Student’s t-test; p < 0.01) (Fig 5). Specifically, the non-human versions of miR-299-3p and miR-541-3p were four and one and a half times more expressed than the human miRNAs respectively, whereas human miR-508-3p was seven times more expressed than the non-human version. Finally, miR-503-3p, which was the only miRNA with one nucleotide substitution in the seed region, had similar expression levels for both versions. Given that the entire precursor molecules were cloned in expression vectors for measuring expression changes, we cannot exclude the contribution of other sources of variation apart from nucleotide changes in the mature region.

**Fig 6:**
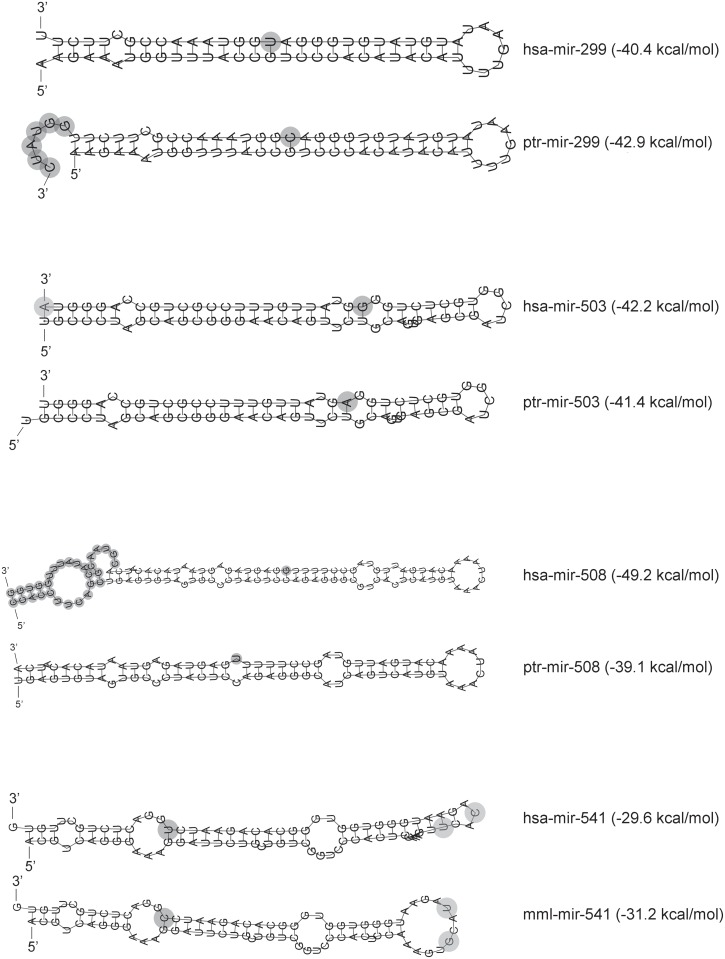
Hairpin structures and stabilities of the studied miRNAs. Minimum free energy (MFE) and secondary structure predictions for human (hsa), chimpanzee (ptr) and macaque (mml) miRNA precursor sequences (miRBase, release 21), according to RNAfold. Grey circles represent nucleotide changes between miRNAs.

Differences in expression levels between versions of miRNAs could be also determined by differences in their molecular stabilities. To address this, we calculated the minimum free energy (MFE) of the human and non-human predicted pre-miRNA secondary structures using RNAfold (Fig 6). Interestingly, MFE differences between the human and non-human hairpins of mir-299, mir-508 and mir-541 were in accordance with the differential expression levels observed between variants: more expressed variants showed higher stabilities and pairs of miRNA variants with stronger expression differences showed larger MFE differences. Besides nucleotide substitutions, precursor molecules of the human and non-human versions of mir-299 and mir-508 described in miRBase also differ in length (Fig 6). To assess the effect of differential precursor lengths in the hairpin stabilities for these two miRNAs, we recalculated MFE values considering only nucleotide substitutions but without considering miRNA length differences (S5 Table). Differences in MFE remained but were lower than when considering the described full length pre-miRNAs. Thus, we conclude that differences in the expression levels between human and non-human miRNAs could be explained by both, human-specific nucleotide substitutions and length differences of the pre-miRNA.

### miRNA variants differentially affect gene expression and regulatory pathways

**Fig 7:**
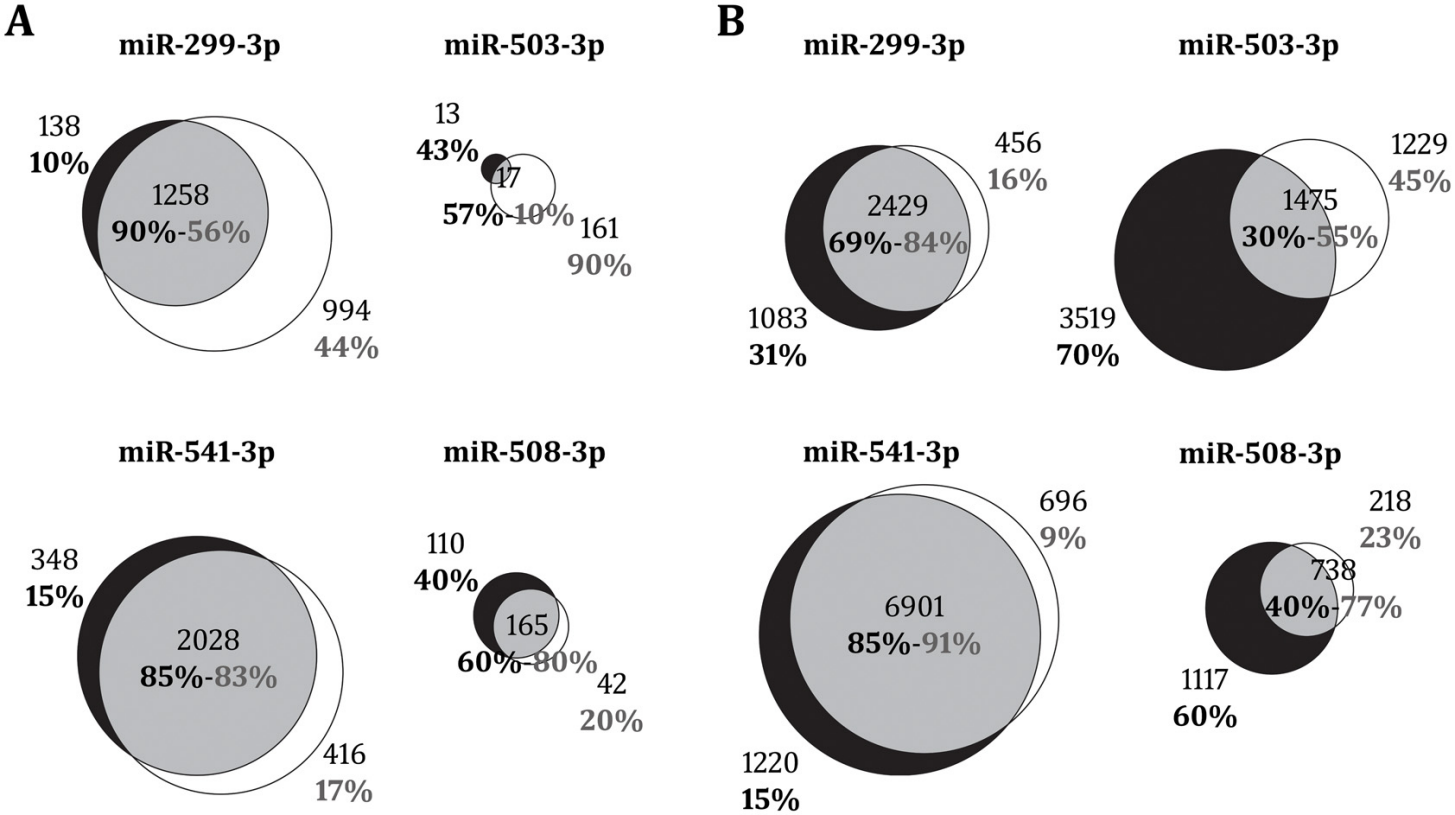
Deregulated and target genes of the studied miRNAs. (**A**) Number of deregulated genes for human (black), non-human (white) and both (grey) miRNA variants from microarray experiments in transfected SH-SY5Y cells. Deregulated genes were obtained based on the fold change in gene expression (adjusted p < 0.05; fold change > 1.2) when comparing cells transfected with the studied miRNA mimic variants and cells transfected with the negative control. (**B**) Number of target genes predicted by PITA algorithm (ΔΔG score ≤ -10) for the studied miRNAs for human (black), non-human (white) and both (grey) miRNA variants. Percentages indicate the portion of exclusive (outside the intersection) or common (inside the intersection) genes among the total number of regulated (**A**) or target (**B**) genes for each variant.

Apart from differences in expression levels, nucleotide substitutions in the mature region could change the spectrum of miRNA target genes, thus we investigated which genes could be regulated by each miRNA variant thorough miRNA overexpression experiments. SH-SY5Y neuroblastoma cells were singled transfected with miRNA mimics of either the human, the non-human primate version of each mature miRNA or a negative control. All four miRNAs modified the expression of a variable number of genes (Moderated t-test; adjusted p < 0.05; fold change > 1.2) (Fig 7A). The human and non-human versions of miR-299-3p and miR-541-3p deregulated more than one thousand genes, while both versions of miR-503-3p and miR-508-3p deregulated less than 300 genes. Interestingly, the proportion of genes exclusively deregulated by human and non-human miRNAs was also very different. Human miR-299-3p and miR-541-3p deregulated about the 10% of the genes in an exclusive manner, but these proportions reached about 50% for human miR-503-3p and miR-508-3p. In the case of non-human miRNAs, the set of exclusively deregulated genes varied for all four miRNAs, from 17% (miR-541-3p) up to 90% (miR-503-3p). We next analyzed the canonical pathways and biological functions associated with the set of genes deregulated by each miRNA variant using the Ingenuity pathway analysis software. Except for miR-503-3p, both the human and non-human versions of each miRNA were involved in similar biological functions, mainly related with cell cycle processes such as proliferation, cell growth or apoptosis (S6 Table). Moreover, genes deregulated by miR-299-3p and miR-541-3p were involved in reproductive and infectious diseases and non-human miR-503-3p, human miR-508-3p and both variants of miR-541-3p were associated with neurological disorders. Remarkably, non-human miR-299-3p regulated genes with important roles in nervous system development and function. As anticipated, miR-503-3p showed the highest variation between variants with human miR-503-3p being particularly involved in the regulation of cancer related signaling pathways and miR-503-3p in metabolic functions.

We finally predicted target genes for the studied miRNAs using PITA algorithm (S7 Table). As expected, the proportion of predicted target genes in common between the human and non-human variants was larger for the three miRNAs with nucleotide substitutions in the mature out of the seed region than for miR-503-3p with the nucleotide substitution in the seed (Fig 7B). This was particularly strong in the case of miR-541-3p for which 85% of the target genes predicted for human miR-541-3p were also predicted for non-human miR-541-3p. In contrast, only 30% of the target genes predicted for the human miR-503-3p were also predicted for the non-human miR-503-3p. Interestingly, we found that, for all four miRNAs, the set of genes exclusively deregulated by either the human or non-human miRNAs were enriched in predicted target genes for the same variant (Chi-square test; p < 0.05). This result may indicate the existence of positive feedbacks in gene regulation in a mechanism in which genes that are directly targeted by the studied miRNAs are, at the same time, involved in regulatory loops that reinforce the activity of these miRNAs. We then looked for genes that were deregulated in the microarray by one of the two miRNA variants and, at the same time, were predicted to be targeted by the same variant (S8 Table). We found of special interest the differences observed between the human and non-human variants of miR-541-3p with the human version potentially regulating genes expressed in the nervous system such as *KCTD11*, *SLC12A6*, and *PMP22* (Table 2). These results, together with the fact that the networks deregulated by miR-541-3p are related to neurological disorders, suggest that molecular changes in this miRNA could have had a role in species-specific phenotypes related to nervous system development and function.

**Table 2:** Candidate genes exclusively deregulated and targeted by human miR-541-3p.

		Microarray gene expression^a^			PITA target gene predictions	
Gene	Gene function	Fold change	Nominal p-value	Adjusted p-value	minimum ΔΔG human variant	minimum ΔΔG non-human variant
*KCTD11*	Potassium channel domain implicated in neuronal differentiation and tumor repression	-1.4	2.32E-03	4.12E-02	-15.6	-11.41
*SLC12A6*	K-Cl transporter involved in electrochemical equilibrium	-1.3	2.88E-03	4.82E-02	-14.46	-9.3
*PMP22*	Membrane protein component of myelin in peripheral nervous system	1.3	1.68E-03	3.24E-02	-13.04	-8.28
*UQCC*	Transmembrane protein involved in fibroblast growth factor regulation	1.3	1.31E-04	4.54E-03	-17.34	-2.17
*TMEM183B*	Transmembrane protein involved in cell interactions	1.2	1.03E-03	2.25E-02	-13.05	-9.35

^a^Deregulated genes by the human miRNA compared with the negative control.

## Discussion

Genome-wide approaches involving sequencing data from different individuals and lineages may help to understand the evolution of regulatory elements such as miRNAs. Nevertheless, this kind of effort must be supported by functional studies in order to explore the real biological relevance of the evolutionary changes. In this work, we took advantage of a large data set of genomic high-coverage sequences from different great apes in the hope of determining sequence variation of putative orthologous great ape miRNAs both intra- and inter-species. We further constructed a catalogue of species-specific nucleotide substitutions and focused in the analysis of four miRNAs with human-specific substitutions showing that these changes affect their regulatory function.

Within the miRNA sequence, the seed has been considered the most conserved miRNA region and the most important domain for miRNA regulatory function. Nevertheless, high conservation has also been reported in certain positions in the mature region in metazoans [10] and human populations [27]. Contributing to extend previous studies, we show, for the first time, similar patterns of conservation between the seed and the entire mature region, highlighting the importance of both domains in the regulatory function of miRNAs. In addition, we observed a strong relationship between miRNA sequence conservation and molecular age, so that younger miRNAs are generally less conserved than older miRNAs. Moreover younger miRNAs tend to be isolated (not clustered, nor duplicated), poorly associated with diseases and less expressed than older miRNAs. Patterns observed in this work would be in line with previous studies showing that low expressed miRNAs are less conserved in primates and *Drosophila* [42,43]; and tend to be younger across mammals [16,17,30].

Understanding the main features that correlate with miRNA conservation may give us insights about which miRNAs may have participated in the evolutionary and speciation processes. Consequently, we created an accurate catalogue of species-specific nucleotide changes in miRNA genes along the great ape phylogeny that could help in deciphering the role of miRNAs in the recent evolution of our species. Focusing on miRNAs with human-specific substitutions and comparing their expression patterns with those of more conserved miRNAs in great apes, we observed that the first are less expressed in certain tissues such as cerebellum, heart and kidney. More specifically, miRNAs with human-specific substitutions located only in the mature region presented a tendency to be lowly expressed in brain. Conversely, we observed no differences in testis. This could be in accordance with Meunier and collaborators, who showed that recent miRNAs are predominantly expressed in testis and suggest testis as a tissue for the trial and establishment of recently emerged non-coding RNAs [16]. We next centered our interest in the analysis of the expression of four miRNAs with human-specific substitutions located either in the mature (miR-299-3p, miR-508-3p and miR-541-3p) or in the seed regions (miR-503-3p). Interestingly, we observed significant differences in the expression levels between human and non-human variants for the first three miRNAs. These expression differences could be attributed to the nucleotide substitutions in the mature region if they change the secondary structure. Changes in the latter could affect miRNA biogenesis if they modify structural requirements that alter the binding efficiency of protein complexes involved in miRNA maturation as the Drosha-DGCR8 or if they reduce the stability and/or capacity of pre-miRNA export to the cytoplasm, which ultimately would decrease the proportion of mature molecules as described for certain human miRNA polymorphisms [44,45]. In addition, changes in the hairpin stabilities of mir-299 and mir-508 could also be affected by changes in the precursor molecule lengths. This may be particularly relevant for mir-508 whose human and non-human hairpins showed the highest significant differences in length and free energy values, as well in their levels of expression.

We also observed differences in the number of genes and networks that the four studied miRNAs regulate. On one hand, miR-299-3p and miR-541-3p change the expression of a large number of genes (between one and two thousands genes). These two miRNAs are located within a large cluster in the human chromosome 14 that was originated before the radiation of modern placental mammals [46]. miRNAs from this cluster are highly expressed in adult brain regions and their target genes are mostly involved in neurogenesis and other neural functions [13,46,47]. In particular, mir-541 is known to play an essential role in neuron-cell proliferation and neurite outgrowth [48]. In accordance we observed a significant expression of miR-299-3p and miR-541-3p in brain tissues from different primates. Moreover, we found that the human version of miR-541-3p may directly regulate several genes involved in nervous system activity such as *KCTD11*, a cell membrane protein expressed in brain that regulates neuronal differentiation [49]; *SLC12A6*, a potassium-chloride cotransporter that has been involved in neuronal disorders [50] and *PMP22*, which is a major component of myelin highly expressed in the peripheral nervous system [51]. Our results suggest that both miRNAs may carry out important functions in the nervous system in humans that could differ from those carried out in other great apes. Conversely, we found that miR-503-3p and miR-508-3p were absent or lowly expressed in the brain compared to other tissues such as testis and placenta. Both genes are located in clusters in the X chromosome and change the expression of a lower number of genes (from 30 to 275) compared to the previous two miRNAs. miR-503 is located together with mir-424 [52], in a cluster that is conserved across mammals [53]. Although specific expression patterns of mir-503 have been poorly investigated it has been shown to be lowly expressed in human brain cortex [53], in accordance with our analyses. In fact, miR-503-3p seems to have a restricted pattern, considering that we only detected its expression in human placenta and macaque testis. Interestingly, two genes typically expressed in placenta and involved in signal transduction and development (*EPS8* and *ZNF644*) were deregulated by the non-human miR-503-3p in the transcriptome experiments and predicted as candidate targets for the same variant (S7 Table). As expected, we found strong differences in the exclusive set of target genes between miR-503-3p variants as they differ in the seed region. These differences are also reflected in the gene networks predicted for each variant as the human miR-503 is involved in cancer signaling pathways while the non-human miR-503 is involved in metabolic functions. On the other hand mir-508 is located in a cluster that is conserved across mammals and it has rapidly evolved in primates [54]. This cluster comprises around 15 miRNAs that are preferentially expressed in testis and are involved in male sexual maturation [19,54]. This is in concordance with the high expression of miR-508-3p that we observed in different primate testis. Interestingly, we detected expression of miR-508-3p in the human brain but not in the other primate brains analyzed. Moreover, miR-508-3p showed the highest variation between variants in the levels of expression being the human variant seven times more expressed than the non-human one. The substantial increase in expression levels of miR-508-3p in the human lineage may have allowed this miRNA to expand its expression to tissues other that testis. Despite the increase in expression levels, we did not detect significant differences at the level of gene networks between the human and non-human variants, as both were mainly involved in apoptosis and development processes. However, by increasing expression in human tissues miR-508-3p may have access to a completely new repertoire of tissue-specific regulatory pathways in humans that would not have been detected in our analysis.

In general, miRNAs can appear *de novo* or they can undergo a neo-functionalization process from existing miRNAs by mutation. We speculate that other putative mechanisms could give rise to changes in miRNA function such as differential biogenesis giving precursor molecules of different lengths among species. We suggest that this could be the case for mir-299 and mir-508 for which the hairpin length between humans and the other great apes has been described to be different. Gene duplication is another common mechanism responsible of the birth of most of the miRNAs [14] as it has been the case for the mir-508 cluster. In addition, functionalization of the minor strand (usually the 3p strand) can generate new mature miRNA molecules and the proportion of functional 5p and 3p strands can vary for the same miRNA within two different species by arm switching [8,55]. Remarkably, all four miRNAs here studied are 3p strands, and all except miR-541-3p are described as the minor strand. This fact could indicate the four studied miRNAs, and probably other miRNAs with human-specific substitutions, are recently established mature miRNAs, which first emerged as part of their precursor molecules that later became functional 3p strands.

We engaged in a deep study of the selective pressures acting on miRNAs molecules to elucidate the functional relevance that certain species-specific modifications may have had in shaping phenotypic differences among species. We find that the whole mature region and not particularly the seed region is under selective constrain, highlighting the importance of the entire mature domain in the miRNA function. We have thoroughly studied the possible functional consequences of human-specific substitutions in the mature region of four miRNAs and show that miR-299-3p and miR-541-3p are conserved miRNAs with neuronal functions and thus evolutionary changes in these miRNAs may have had an impact in gene regulatory networks ultimately related to the evolution of the nervous system. Conversely, miR-503-3p and miR-508-3p regulate a low number of genes, have a restrictive pattern of expression and, although they seem to be involved in development, their functional role is still very vague. We show that specific nucleotide substitutions in the mature region and/or changes in the length of existing miRNAs may affect miRNA expression and hypothesize that both could be mechanisms by which miRNAs acquire new regulatory functions. Our study addresses for the first time the role that human-specific substitutions in miRNAs could have had in our recent evolutionary history and enlarges our understanding of how the non-coding genome may have contributed to shape human-specific traits.

## Supporting Information

S1 Fig Distribution of miRNAs in the PCA analysis. (PDF)

S1 Table Number of individuals in each great ape population used in this study. Information based on Prado-Martinez et al. (2013).
(PDF)

S2 Table Primers used for miRNA cloning and qPCR expression analysis. (PDF)

S3 Table Conservation among miRNA regions. Top right in grey: p-values for the generalized linear model used to compare conservation among different miRNA regions. The model takes into account GC content: SNV density ~ GC content + miRNA region. Bottom left in white: p-values for the paired t-test comparing concatenated nucleotide diversity values of each miRNA region. Asterisks indicate p-value < 0.05.
(PDF)

S4 Table Main characteristics of the 235 miRNAs carrying human-specific nucleotide substitutions. (XLS)

S5 Table Minimum free energy values (MFE). MFE in kcal/mol of the secondary structures predicted for the four studied miRNAs according to RNAfold.
(PDF)

S6 Table Top significant pathways and networks associated with genes exclusively deregulated by the human or the non-human miRNAs. Data based on the Ingenuity Pathway Analysis software.
(PDF)

S7 Table Target genes predicted by PITA for each miRNA. (XLS)

S8 Table Candidate target genes exclusively deregulated by one of the two miRNA variants and predicted by PITA with high confidence for that variant. (XLS)
