# Supplementary material for: Functional Implications of Human-Specific Changes in Great Ape microRNAs
Source: PLoS One. 2016 Apr 22;11(4):e0154194. doi: 10.1371/journal.pone.0154194 (PMC4841587; doi:10.1371/journal.pone.0154194)
Supplement: S1 Fig — (PDF) [file pone.0154194.s001.pdf]

S1 Figure. Distribution of miRNAs in the PCA analysis. Each plot corresponds to one variable used on the PCA analysis. Molecular age is taken from Zhu et al. [1] and Iwama et al. [2] where each integer represents a period of origin. In [1] oldest miRNAs have the highest values while in [2] oldest miRNAs have the lowest values. Number of diseases explains the first component and molecular age the second component. Conserved miRNAs (low SNV density values) tend to be involved in a larger number of diseases, older and located in clusters.

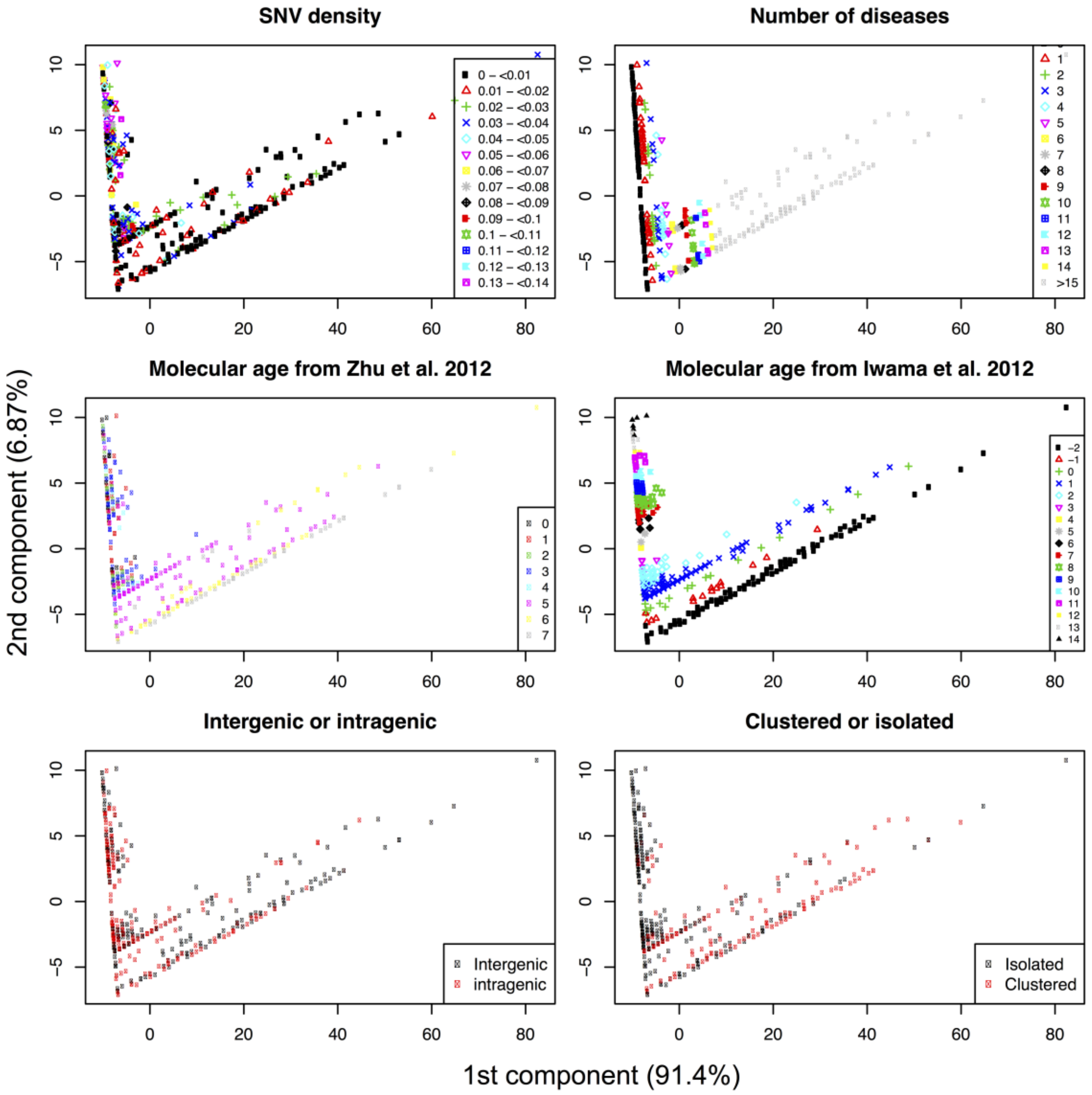

1. Zhu Y, Skogerbø G, Ning Q, Wang Z, Li B, Yang S, et al. Evolutionary relationships between miRNA genes and their activity. BMC Genomics. 2012;13: 718. doi:10.1186/1471-2164-13-718

2. Iwama H, Kato K, Imachi H, Murao K, Masaki T. Human microRNAs originated from two periods at accelerated rates in mammalian evolution. Mol Biol Evol. 2013;30: 613–26. doi:10.1093/molbev/mss262
