# Supplementary material for: Functional Implications of Human-Specific Changes in Great Ape microRNAs
Source: PLoS One. 2016 Apr 22;11(4):e0154194. doi: 10.1371/journal.pone.0154194 (PMC4841587; doi:10.1371/journal.pone.0154194)
Supplement: S1 Table — Information based on Prado-Martinez et al. (2013). (PDF) [file pone.0154194.s002.pdf]

S1 Table. Number of individuals in each great ape population used in this study. Information based on Prado-Martinez et al. [1].

Common name	Species name	Number of individuals
Human	*Homo sapiens*	9
Chimpanzee	*Pan troglodytes verus*	4
	Pan troglodytes schweinfurthii	6
	Pan troglodytes troglodytes	4
	Pan troglodytes ellioti	10
Bonobo	*Pan paniscus*	13
Gorilla	*Gorilla gorilla gorilla*	23
	Gorilla beringei graueri	3
Orangutan	*Pongo abelii*	5
	Pongo pygmaeus	5
Total	Total	82

1. Prado-Martinez J, Sudmant PH, Kidd JM, Li H, Kelley JL, Lorente-Galdos B, et al. Great ape genetic diversity and population history. Nature. 2013;499: 471–5. doi:10.1038/nature12228
