# Supplementary material for: Functional Implications of Human-Specific Changes in Great Ape microRNAs
Source: PLoS One. 2016 Apr 22;11(4):e0154194. doi: 10.1371/journal.pone.0154194 (PMC4841587; doi:10.1371/journal.pone.0154194)
Supplement: S2 Table — (PDF) [file pone.0154194.s003.pdf]

S2 Table. Primers used for miRNA cloning and qPCR expression analysis.

miRNA	Primer sequence	Amplified fragment size	Use
mir-299	Fw: ACACACACCTCGAGAGTTCTGCCTTGGGAAATCG<br>Rv: ACACACACGGATCCTGTTTGGCACTGAGGAGAAG	489 bp [a]	miRNA cloning
mir-541	Fw: ACACACACCTCGAGGTTCCACTCCTGCTGACTCC<br>Rv: ACACACACGGATCCTATCAACAGCTGGCTGACCA	554 bp [a]	miRNA cloning
mir-508	Fw: ACACACACGGATCCGCATGCCTCCTTTGGACAAG<br>Rv: ACACACACCTCGAGATTTGTTGCCGACTGAAGAGA	511 bp [a]	miRNA cloning
mir-503	Fw: ACACACACGGATCCGGGGTGCCAAAATGAAGCAT<br>Rv: ACACACACCTCGAGGCTCGCGTCTCTGTTTCC	395 bp [a]	miRNA cloning
mir-938	Fw: CACACACAAGATCTCCAAATCATTCTGGCAGTGA<br>Rv: CACACACACTGCAGTTCATTGCTTGTTGGGATCA	549 bp [a]	miRNA cloning
hsa-miR-299-3p	Fw: AGTATGTGGGA**T**GGTAAACC<br>Rv: GGTCCAGTTTTTTTTTTTTTTTAAGC	46 bp	qPCR analyses
ptr-miR-299-3p	Fw: GTATGTGGGA**C**GGTAAACC<br>Rv: GGTCCAGTTTTTTTTTTTTTTTAAGC	45 bp	qPCR analyses
hsa-miR-503-3p	Fw: CGCAGGG**G**GTATTGTTTC<br>Rv: GTTTTTTTTTTTTTTTCCTGGCA	44 bp	qPCR analyses
ptr miR-503-3p	Fw: CGCAGGG**A**GTATTGTTTC<br>Rv: GTTTTTTTTTTTTTTTCCTGGCA	44 bp	qPCR analyses
hsa-miR-508-3p	Fw: CAGTGATTGTAGCCTTTT**G**GA<br>Rv: AGGTCCAGTTTTTTTTTTTTTTTCTAC	48 bp	qPCR analyses
ptr-miR-508-3p	Fw: GCAGTGATTGTAGCCTTTT**T**GA<br>Rv: AGGTCCAGTTTTTTTTTTTTTTTCTAC	49 bp	qPCR analyses
hsa-miR-541-3p<br>mml-miR-541-3p[b]	Fw: CAGTGGTGGGCACAGA<br>Rv: GTCCAGTTTTTTTTTTTTTTTAGTCC	46 bp	qPCR analyses
hsa-miR-938-5p	Fw: CAGTGCCCTTAAAGGTGA<br>Rv: CAGTTTTTTTTTTTTTTTACTGGGT	43 bp	qPCR analyses

Nucleotides shown in bold letters indicate the difference between the human and non-human miRNA variants. [a]The cloning fragment size includes the precursor miRNA sequence plus 120-300 bp at both extremes and primers tail recognized by the restriction enzyme. [b]Non-human variants were chimpanzee miRNA sequences, except miR-541-3p, for which we used the macaque sequence due to the pre-miRNA has not been described in chimpanzee. Same primers were used for human and macaque miR-541-3p qPCR amplification since they did not match the nucleotide substitution between both variants.
