# Supplementary material for: Functional Implications of Human-Specific Changes in Great Ape microRNAs
Source: PLoS One. 2016 Apr 22;11(4):e0154194. doi: 10.1371/journal.pone.0154194 (PMC4841587; doi:10.1371/journal.pone.0154194)
Supplement: S3 Table — Top right in grey: p-values for the generalized linear model used to compare conservation among different miRNA regions. The model takes into account GC content: SNV density ~ GC content + miRNA region. Bottom left in white: p-values for the paired t-test comparing concatenated nucleotide diversity values of each miRNA region. Asterisks indicate p-value < 0.05. (PDF) [file pone.0154194.s004.pdf]

S3 Table. Conservation among miRNA regions. Top right in grey: p-values for the generalized linear model used to compare conservation among different miRNA regions. The model takes into account GC content: SNV density ~ GC content + miRNA region. Bottom left in white: p-values for the paired t-test comparing concatenated nucleotide diversity values of each miRNA region. Asterisks indicate p-value < 0.05.

	Flanking 3’	Flanking 5’	Precursor miRNA	Mature miRNA	Seed region
Flanking 3’		0.678809	0.029605*	2.75E-05*	0.000151*
Flanking 5’	0.08245		0.00845*	4.36E-06*	2.79E-05*
Precursor miRNA	8.892E-05*	8.979E-08*		0.00867*	0.030776*
Mature miRNA	1.331E-07*	6.64E-07*	0.005981*		0.69483
Seed region	6.203E-05*	1.149E-06*	0.007482*	0.5417	
