# Supplementary material for: Functional Implications of Human-Specific Changes in Great Ape microRNAs
Source: PLoS One. 2016 Apr 22;11(4):e0154194. doi: 10.1371/journal.pone.0154194 (PMC4841587; doi:10.1371/journal.pone.0154194)
Supplement: S5 Table — MFE in kcal/mol of the secondary structures predicted for the four studied miRNAs according to RNAfold. (PDF) [file pone.0154194.s006.pdf]

**S5 Table. Minimum free energy values (MFE).** MFE values in kcal/mol of the secondary structures predicted for the four studied miRNAs according to RNAfold.

| | MFE human variant | MFE non-human variant | MFE non-human variant only considering nucleotide substitutions in 3p strand |
|---|---|---|---|
| mir-299 | -40.4 | -42.9 | -42.2 |
| mir-503 | -42.2 | -41.4 | -42.2 |
| mir-508 | -49.2 | -39.1 | -42.6 |
| mir-541 | -29.6 | -31.2 | -31.5 |
