# Supplementary material for: Functional Implications of Human-Specific Changes in Great Ape microRNAs
Source: PLoS One. 2016 Apr 22;11(4):e0154194. doi: 10.1371/journal.pone.0154194 (PMC4841587; doi:10.1371/journal.pone.0154194)
Supplement: S6 Table — Data based on the Ingenuity Pathway Analysis software. (PDF) [file pone.0154194.s007.pdf]

Table S6. Top significant pathways and networks associated with genes exclusively deregulated by the human or the non-human miRNAs. Data based on the Ingenuity Pathway Analysis software.

		Top 5 canonical pathways	Top 5 networks
miR-299-3p	Human	RAN Signaling<br>Estrogen-mediated S-phase Entry<br>Ethanol Degradation IV<br>Antiproliferative Role of TOB in T Cell Signaling<br>Remodeling of Epithelial Adherens Junctions	Carbohydrate Metabolism, Hereditary Disorder, Lipid Metabolism<br>Carbohydrate Metabolism, Small Molecule Biochemistry, Cell Cycle<br>Cell Morphology, Cellular Compromise, Developmental Disorder<br>RNA Post-Transcriptional Modification, Small Molecule Biochemistry, Hereditary Disorder<br>Cellular Assembly and Organization, Cardiovascular Disease, Cardiovascular System Development and Function
	Non-human	RAN Signaling<br>Estrogen-mediated S-phase Entry<br>Protein Ubiquitination Pathway<br>Mitochondrial Dysfunction<br>Aryl Hydrocarbon Receptor Signaling	Hereditary Disorder, Metabolic Disease, Developmental Disorder<br>Connective Tissue Disorders, Developmental Disorder, Hereditary Disorder<br>RNA Post-Transcriptional Modification, Infectious Disease, Cardiovascular System Development and Function<br>RNA Post-Transcriptional Modification, Developmental Disorder, Hereditary Disorder<br>Cellular Assembly and Organization, Nervous System Development and Function, Cancer
miR-503-3p	Human	Melanoma Signaling<br>ERK/MAPK Signaling<br>Breast Cancer Regulation by Stathmin1<br>Regulation of Cellular Mechanics by Calpain Protease<br>Antiproliferative Role of Somatostatin Receptor	Cell Cycle, Small Molecule Biochemistry, Cell-mediated Immune Response<br>Digestive System Development and Function, Hepatic System Development and Function, Organ Development<br>Post-Translational Modification, Cell Signaling, Gene Expression
	Non-human	Cardiac b-adrenergic Signaling<br>ERK/MAPK Signaling<br>FLT3 Signaling in Hematopoietic Progenitor Cells<br>Ubiquinol-10 Biosynthesis (Eukaryotic)<br>Insulin Receptor Signaling	Cell Morphology, Cellular Compromise, Cellular Assembly and Organization<br>Cell Death and Survival, Cell Morphology, Cellular Assembly and Organization<br>Increased Levels of Albumin, Cellular Assembly and Organization, RNA Post-Transcriptional Modification<br>Cancer, Immunological Disease, Organismal Injury and Abnormalities<br>RNA Post-Transcriptional Modification, Developmental Disorder, Neurological Disease

miR-508-3p	Human	Apoptosis Signaling<br>PI3K/AKT Signaling<br>PEDF Signaling<br>TWEAK Signaling<br>PTEN Signaling	Cancer, Embryonic Development, Cardiac Regeneration<br>Cell Cycle, Cell Morphology, Cellular Function and Maintenance<br>Cellular Movement, Hematological System Development and Function, Immune Cell Trafficking<br>Cellular Assembly and Organization, Cellular Function and Maintenance, Protein Synthesis<br>Cancer, Neurological Disease, Cell Morphology
	Non-human	Apoptosis Signaling<br>TNFR1 Signaling<br>CD27 Signaling in Lymphocytes<br>TWEAK Signaling<br>PI3K/AKT Signaling	Cellular Response to Therapeutics, Connective Tissue Development and Function, Cell Cycle<br>Molecular Transport, Cellular Assembly and Organization, Tissue Development<br>Cell Death and Survival, Cardiovascular System Development and Function, Organ Morphology<br>Cellular Assembly and Organization, Cellular Function and Maintenance, Cell Morphology<br>Cellular Assembly and Organization, Cellular Function and Maintenance, Cell Cycle
miR-541-3p	Human	NRF2-mediated Oxidative Stress Response<br>PI3K/AKT Signaling<br>Acute Myeloid Leukemia Signaling<br>Protein Ubiquitination Pathway<br>Estrogen Receptor Signaling	Hereditary Disorder, Neurological Disease, Psychological Disorders<br>Cellular Assembly and Organization, Cell Morphology, Cellular Function and Maintenance<br>Gene Expression, Gastrointestinal Disease, Cell Death and Survival<br>Developmental Disorder, Hereditary Disorder, Metabolic Disease<br>RNA Post-Transcriptional Modification, Cancer, Tumor Morphology
	Non-human	NRF2-mediated Oxidative Stress Response<br>Protein Ubiquitination Pathway<br>Mitochondrial Dysfunction<br>Acute Myeloid Leukemia Signaling<br>Pyridoxal 5'-phosphate Salvage Pathway	Gene Expression, Carbohydrate Metabolism, Developmental Disorder<br>Dermatological Diseases and Conditions, Developmental Disorder, Endocrine System Disorders<br>Developmental Disorder, Hereditary Disorder, Neurological Disease<br>Cellular Development, Cellular Growth and Proliferation, Developmental Disorder<br>Cell-To-Cell Signaling and Interaction, Hematological System Development and Function, Tissue Development
